# Structural drivers of health inequality in sub-Saharan Africa: Evidence and policy implications

**DOI:** 10.1016/j.hpopen.2025.100151

**Published:** 2025-11-05

**Authors:** Mercedes Tejería-Martínez, Vanesa Jordá, José María Sarabia

**Affiliations:** Department of Economics, University of Cantabria, Spain

**Keywords:** Inequality, Health, Body mass index, Conditional inference trees, Sub-Saharan Africa

## Abstract

Health inequalities remain a major challenge in global development, particularly in sub-Saharan Africa (SSA), where disparities are exacerbated by limited access to healthcare and widespread socioeconomic inequities. This study examines health inequality in 10 SSA countries using Body Mass Index as a health outcome. Drawing on data from the Demographic and Health Surveys, we employ conditional inference trees to assess the extent of health disparities by classifying populations into groups based on shared socioeconomic status, demographic characteristics, and lifestyle choices. Our analysis reveals significant health inequality, particularly in Mauritania, Eswatini, and Kenya, while Egypt emerges as the most equal country in terms of health outcomes. Furthermore, our findings show that disparities are largely driven by illegitimate sources of inequality, such as wealth and education, while legitimate factors linked to personal decisions have a minimal impact. Demographic factors, particularly age, are the largest contributors to health disparities in most countries, with gender also standing as a major determinant in many countries. These findings underscore the need for targeted health policies that address the root causes of inequality, such as expanding access to healthcare, implementing social protection programs, and promoting gender equality in health.

## Highlights

• This paper presents a comprehensive analysis of health disparities in 10 SSA countries.
• We focus on both men and women, using BMI as a health indicator.
• Socioeconomic factors drive disparities, while lifestyle choices have minimal impact.
• We find notable gender disparities, suggesting systemic discrimination in health access.
• Policy implications underscore the need to combat health disparities by focusing on marginalized populations.

## 1 Introduction

Health disparities have been a central focus of government policies for the past 40 years. The Black Report, first published in the 1980s, underscored the growing importance of measuring health inequalities and identifying the root causes of differences in health outcomes [1]. Numerous studies have since explored the relationship between health and a range of socioeconomic variables as well as genetic factors (e.g., [2,3,4]). This body of research consistently identifies income, region-specific cultural behaviors, and exogenous factors beyond individuals’ control – such as parental wealth and education – as key drivers of health inequalities [5].

A potential limitation in health inequality research is its tendency to focus on high-income countries, particularly in Europe, the United States, Canada, and Australia (see e.g., [6,7,8,2,4])^1^ Yet disparities are likely to be more severe in low- and middle-income countries, especially in sub-Saharan Africa (SSA). Many SSA countries face extreme health challenges, including high rates of undernutrition, widespread infectious diseases [9], and limited health insurance coverage, with only 8 of 36 SSA countries reporting coverage above 10 percent [10].

The challenge of obtaining comprehensive health data is exacerbated by ongoing debates over how to accurately measure individuals’ health status [11]. Much of the existing research on health inequality has focused on subjective measurements such as self-assessed health (SAH) (e.g., [7,11]). However, SAH has notable limitations, particularly its susceptibility to individual perceptions, which may not accurately reflect actual health conditions [12,13]. Furthermore, prior evidence suggests that SAH measurements are affected by systematic misreporting biases that vary with socioeconomic status and educational attainment [14,2].

Despite these limitations of SAH, few analyses have assessed health disparities using objective measurements, particularly in SSA. Where such evaluations exist, they have predominantly focused on anthropometric measurements, for example, the height-for-age Z-score [15,16]. While effective for assessing malnutrition, these metrics fail to capture the complete spectrum of health challenges. Notably, obesity has emerged as a significant concern in SSA, a key risk factor for non-communicable diseases that now constitutes nearly 30 percent of the region’s total disease burden [17].

This study aims to fill a critical gap in the literature by providing a comprehensive analysis of health disparities in the SSA region for both men and women. Much of the existing research in low- and middle-income countries has focused on health disparities among children or women, while adult men have received comparatively little attention. We examine health inequalities in 10 countries in SSA using the Body Mass Index (BMI) as an indicator of health status, based on nationally representative data from the Demographic and Health Surveys (2022) [18]. BMI provides valuable information for monitoring both malnutrition and obesity prevalence, and serves as a predictive measurement for multiple metabolic risks and nutritional status.

However, BMI is not without limitations. Errors in its calculation often result from inaccuracies in reported height and weight. Fortunately, these measurement errors are typically random and do not systematically bias estimates [19]. Additionally, BMI increases substantially during the first 20 years of life, but stabilizes in early adulthood [20] and remains relatively steady until around 55 years of age [21]. We focus our analysis on adults aged 20 to 55, a period during which BMI is relatively stable to ensure meaningful comparisons. Furthermore, we standardize the BMI to a reference level, facilitating consistent comparisons across different demographic groups [19].^2^

We consider multiple factors that may contribute to unfair and avoidable health disparities, instead of relying on approaches that assess health inequality based on a single source (usually income). This comprehensive perspective acknowledges that health inequality stems from multiple individual endowments rather than income alone and aligns with the World Health Organization’s definition of health inequality, established by the World Health Assembly in 1979. Following the approach proposed by Fleurbaey and Schokkaert [3], we distinguish between legitimate and illegitimate sources of health disparities to accurately assess health inequality. This distinction helps identify the main drivers of inequality and quantify the contribution of different factors to health differences.

Health inequality is straightforward to analyze when only one factor, such as income, is considered. While the multidimensional framework proposed in this study should provide more accurate estimates, incorporating multiple determinants complicates the definition of homogeneous groups. To overcome this challenge, we employ a data-driven approach that systematically partitions the population into well-defined segments, enabling a more nuanced and objective assessment of health disparities.

Our results indicate that while socioeconomic disparities are the most influential factors, legitimate factors – such as personal lifestyle choices – account for only a marginal share of overall disparities. Additionally, demographic characteristics play a significant role, with age and gender emerging as decisive factors. This finding highlights the risk of overlooking health disparities affecting men, as seen in much of the previous literature on health inequality in low- and middle-income countries, leading to an incomplete understanding of the issue.

The remainder of the paper is structured as follows: First, we introduce the methodology for measuring health inequality. We next describe the key characteristics of the DHS data, detailing the health indicators and socioeconomic determinants used to model health inequality. We then present the estimates of inequality in the BMI distribution and provide a decomposition of legitimate and illegitimate sources. Finally, we conclude with a discussion of the study’s main findings and their implications for health policy design.

## 2 Methods

In recent decades, growing concerns about widening health disparities in lower-income countries have led scholars to focus on providing more precise estimates of health inequality. Much of this research focuses on socioeconomic disparities, often framing health inequality in terms of differences between, rather than within, socioeconomic groups. This perspective assumes that inequalities arising from factors beyond an individual’s control are more problematic than those linked to personal choices. The distinction between “legitimate” and “illegitimate” health inequalities is rooted in this reasoning, where the former are seen as consequences of individual responsibility, while the latter stem from structural disadvantages [3].

However, beyond socioeconomic status, numerous other factors contribute to health disparities, raising ethical concerns about the fairness and avoidability of such differences. We consider a broader set of determinants that may influence variations in health outcomes, to provide a more comprehensive perspective. Specifically, we account for socioeconomic status alongside demographic characteristics and lifestyle choices, recognizing that health disparities arise from a complex interplay of structural, behavioral, and biological factors.

Let us assume that an individual’s health level (*h_i_*) can be defined as:

(1) $$h_{i}=f\left(y_{i},d_{i},l_{i},u_{i}\right),i=1,\cdots ,n,$$

where *y_i_* is a vector of variables related to socioeconomic status, *d_i_* represents demographic variables, *l_i_* includes lifestyle-related factors, and *u_i_* is an unobserved error term.

Our approach aligns with the broader theoretical framework of socioeconomic inequality by defining health inequality as differences in health levels between groups of individuals sharing homogeneous characteristics. If $y_{i}$ were a single socioeconomic variable, such as income, health inequality would traditionally be analyzed solely in terms of differences between income groups. However, this conventional approach oversimplifies the complexity of health disparities, which are influenced by multiple interrelated factors. We incorporate a broader range of determinants to address this limitation, providing a more comprehensive perspective on the drivers of health inequality.

Socioeconomic variables, such as wealth and education, are widely regarded as illegitimate sources of health inequality, as they reflect structural disparities rather than individual choices. In contrast, lifestyle choices (e.g., smoking, diet) are often considered legitimate sources of health inequality under the assumption that individuals should bear some responsibility for their health-related decisions.

Demographic variables present a more complex case. Age and sex naturally influence health outcomes due to biological factors, but disparities arising from these characteristics are not entirely neutral. For instance, gender-based health differences may reflect discrimination in access to resources, healthcare, or social norms that disadvantage certain groups. Similarly, age-related disparities can be influenced by unequal access to healthcare, employment opportunities, or social protection. We standardize BMI so that it is normalized to be equivalent to that of a 20-year-old woman, in order to isolate the role of these non-biological factors (see Section 3.1). This adjustment ensures that any remaining differences attributed to age and sex reflect social and structural inequalities, rather than inherent physiological variations.

While our approach incorporates a wide range of determinants, this richer framework complicates the definition of homogeneous groups, as individuals may differ across multiple dimensions simultaneously. To address this challenge and avoid arbitrary choices, we employ a data-driven approach to systematically partition the population into groups with similar characteristics. This data-driven approach enables a more nuanced and objective analysis of health disparities by systematically creating well-defined population segments. By grouping individuals who share meaningful common characteristics, it ensures that health inequalities are assessed in a way that reflects the complex interplay of multiple influencing factors.

We employ conditional inference trees to partition the population into groups with similar socioeconomic positions and homogeneous characteristics. This algorithm recursively selects the variable that best predicts variation in health outcomes to divide the population into non-overlapping groups. These partitions ensure internal homogeneity in individual characteristics and are established through a series of permutation tests, a process known as recursive binary splitting. Specifically, we apply the method developed by Hothorn et al. [22], which begins by testing the null hypothesis of independence for each individual characteristic. The variable with the lowest *p*-value determines the first partition of the population, provided it is statistically significant.

Independence tests are conducted for every possible population partition based on the values of this variable, and the population is divided using the splitting point associated with the lowest p-value. The algorithm keeps running until none of the individual features passes the independence test, indicating that characteristics no longer effectively classify individuals into groups with distinct sets of characteristics. The expected value of *h_i_* is calculated as the average BMI within group *i*.

Although conditional inference trees are useful for avoiding arbitrary decisions in model specification, their estimates can exhibit significant variability. The structure of the tree, including the number of nodes and partitions, is highly sensitive to the first variable selected for splitting [23]. Additionally, cross-country comparisons are complicated by differences in sample sizes, which can vary considerably [24].

To mitigate the impact of sample size on health inequality estimates, we follow Brunori and Neidhöfer [24] and generate 200 random samples of size 1,003,^3^ from which trees are re-estimated. Health inequality estimates are then computed for each of these random samples, and the final estimate is obtained by averaging these estimates across all iterations [25].

Once the population has been partitioned into homogeneous groups, we assess BMI disparities between them. While prior health inequality research often used concentration curves, this approach is limited to single-dimension analysis (typically income-based). Our multidimensional methodology incorporates socioeconomic status, demographics, and lifestyle factors, creating groups without natural hierarchical ordering. Hence, concentration curves are unsuitable for this methodological framework as they require one-dimensional ranking to measure inequality.

To evaluate health inequality, we apply inequality measures to summarize the differences in the average level of health between groups. However, when working with bounded variables such as BMI, relative inequality measures can produce misleading results because proportional changes near the upper and lower limits are harder to achieve. Our baseline results rely on absolute inequality measures to avoid this distortion, as they provide a more accurate representation of disparities by considering actual differences rather than ratios relative to the mean. In particular, we use the variance. To ensure the robustness of our findings, we also consider other widely recognized inequality indices, specifically the Gini index and the mean logarithmic deviation (MLD).

To identify the relative importance of each factor in shaping health disparities, we apply the standard Shapley-Shorrocks value decomposition method [26,27]. This approach quantifies the contribution of each determinant by measuring the reduction in predicted inequality when a specific variable is excluded from the model. The process is repeated across all possible combinations of determinants that exclude the given variable, and the results are averaged to ensure a fair allocation of explanatory power.

## 3 Data

The DHS Program is an initiative responsible for collecting, analyzing, and disseminating health-related data in over 90 countries. DHS data provide reliable information on a wide range of biomarkers across multiple survey waves spanning from 1984 to 2023. The DHS Program, established by USAID in 1984, has provided technical assistance to more than 350 surveys in over 90 countries, implementing standardized protocols that ensure data quality and cross-national comparability [28]. DHS surveys are nationally-representative household surveys that employ a two-stage stratified cluster sampling design, which guarantees that results are generalizable to the population as a whole and comparable across regions and time.

In addition, the program ensures methodological consistency across survey waves, which allows us to examine long-term trends in health and well-being. The credibility of DHS data is further reinforced by strong collaborations with national statistical agencies and ministries of health, ensuring that surveys adhere to international standards while addressing local policy needs. As a result, DHS surveys have become a cornerstone for global health monitoring, policy evaluation, and academic research.

Despite the broad availability of DHS data, our analysis focuses on only 10 countries because not all surveys meet the requirements of our study. To be included, a survey had to meet two criteria: both adult male and female populations were included with sufficiently large samples (at least 1,000 individuals per country), and all variables required for our analysis were available, including BMI measurements and the full set of socioeconomic, demographic, and lifestyle determinants described below.

### 3.1 The BMI

When measuring health inequality, selecting an appropriate outcome variable is crucial. Previous research has employed a variety of biomarkers, such as anthropometric measurements, mortality indicators, and Disability-Adjusted Life Years [15,16,29]. In contrast, our analysis uses BMI, which effectively reflects caloric consumption and overall health [19]. BMI reliably assesses both obesity [30,31,32] and malnutrition [33,34], while also indicating risk for various metabolic conditions. Its comparability across countries makes it particularly valuable as a global health metric [35].

BMI was standardized by age and sex to a reference level to ensure meaningful comparisons across demographic groups prior to analyzing health disparities. This adjustment is crucial because BMI naturally varies with age and differs systematically between men and women due to physiological and metabolic differences. In particular, we follow Sahn and Younger [19] and define the standardized BMI as:

(2) $${\mathrm{BMI}}_{s}=F_{\overset{¯}{a},\overset{¯}{g}}^{-1}\left(F_{a,g}\left(bmi\right)\right),$$

where $\overset{¯}{a}=20,\overset{¯}{g}=female,$ *F* is the cumulative distribution function of the BMI in a reference population of age *a* and gender *g* and *F*^-1^ is the quantile function. Hence, the standardized BMI measurement ensures that an individual’s percentile rank remains consistent between their actual BMI within their age/sex group and their transformed BMI within the reference distribution of 20-year-old females. This guarantees meaningful comparisons across different demographic groups, setting the same standard for all individual BMIs.

### 3.2 Determinants of health inequality

The World Health Organization defines health inequalities as avoidable differences arising from social factors, discrimination, or limited access to essential resources [36]. This analysis examines health disparities by considering two main sources of inequality: illegitimate drivers, such as respondents’ demographic characteristics and socioeconomic status; and legitimate factors, focusing on personal habits through healthy lifestyles such as alcohol drinking, smoking, or a healthy diet. Disparities among groups of individuals sharing similar characteristics in these dimensions help explain the observed inequalities in health outcomes.

We consider demographic factors to be key health determinants. For biological reasons unrelated to health disparities, BMI naturally varies by gender and age. We have restricted our sample to men and women aged 20 to 55 years to minimize the influence of BMI differences attributable to these biological factors [20,21]. Additionally, we have standardized BMI as described in the previous section. These adjustments allow us to isolate the impact of age- and gender-related factors driving health disparities.

In addition to demographic determinants, this analysis considers socioeconomic characteristics as key contributors to health disparities. Socioeconomic status provides a clear snapshot of an individual’s economic background [37]. We use the wealth index provided by the DHS, which serves as a reliable proxy for income or consumption in low-income countries [38], to incorporate this variable into the analysis.

Education, closely tied to socioeconomic status, is another significant factor influencing health outcomes [39]. Recognized as a key determinant of social position, education plays a central role in shaping access to resources and opportunities that impact health. We include the highest number of completed years of education as a determinant in our analysis to capture its variability and potential contribution to health inequality [40,11,41].

Access to basic durable goods is also considered in the analysis. We follow previous literature and include variables indicating whether an individual owns a car, a TV, or a cellphone [42,43,44]. These items are essential for daily life, and a lack of access often reflects financial constraints that prevent individuals from meeting their basic material needs [45,46]. Moreover, in low- and middle-income countries, spending on durable goods is closely linked to financial stability.

An individual’s place of residence, whether rural or urban, is also considered a key determinant of health inequality. Previous research has shown that healthcare expenditures are often a last resort for rural residents, highlighting disparities in access to and use of healthcare services [47]. In recent years, the gap between rural and urban areas has widened, further emphasizing the importance of considering a household’s location when analyzing health inequalities [48,49].

We also consider legitimate contributors to health disparities. Individual behaviors, such as engaging in physical activity, consuming alcohol, and maintaining adequate sleep, are widely recognized as influencing health status [50,51]. In this context, we address the role of personal habits in achieving good health as an essential factor in understanding health disparities. Specifically, tobacco consumption demonstrates a complex relationship with BMI. On average, smokers tend to have a lower BMI; however, among those who already smoke, an increase in the number of cigarettes consumed is associated with a rise in BMI [52]. We include the number of cigarettes smoked daily as a determinant variable in our analysis to account for the impact of tobacco consumption on health disparities.

The DHS also provides data on vegetable consumption among children in the household. While information for adults is unavailable, children’s eating habits are likely to reflect those of their parents. We assume that households providing vegetables for their children are more likely to have healthier eating habits overall. Therefore, we include children’s vegetable consumption (recorded for the day or night before the survey) as a determinant variable in our analysis.

Although alcohol consumption influences BMI [53,54] and is considered a legitimate variable in health inequality research [50,37], we exclude it due to two constraints: potential underreporting in countries with large Muslim populations (see Table A1 in the Appendix) where alcohol is prohibited, and limited data availability in only 7 of 36 sub-Saharan African countries surveyed.

## 4 Results

This section presents our empirical findings on health inequality across 10 sub-Saharan African countries. Our analysis proceeds in two stages. First, we quantify the overall magnitude of health inequality within each country using three complementary inequality measures (the variance, the Gini coefficient, and the MLD) to establish the extent of health disparities. Once inequality has been analysed, we decompose these inequalities to identify the relative contributions of socioeconomic, demographic, and lifestyle factors. This dual approach allows us to both document cross-country variations in health inequality and understand the underlying mechanisms driving these disparities.

### 4.1 Estimating health inequality

**Fig. 1:**
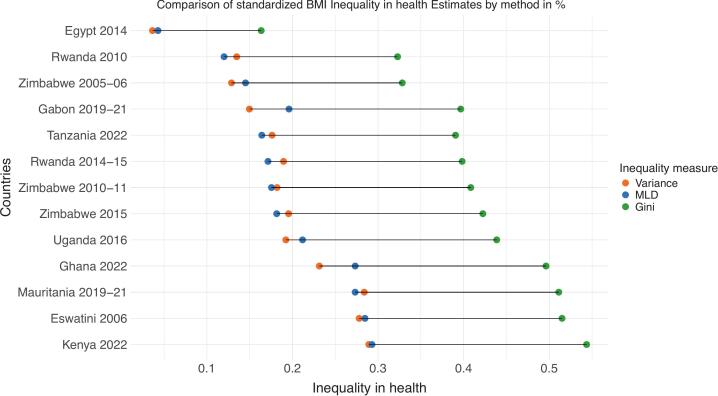
Health inequality in 10 selected SSA (sub-Saharan Africa) countries. .

Source: Authors’ compilation. Note: Health inequality has been estimated using conditional inference-tuned trees, with the optimal parameters detailed in Table A4. Inequality in health is expressed as a proportion of the total variation in BMI (Body Mass Index)

Our analysis begins by examining estimates of health inequality associated with demographic factors, socioeconomic characteristics, and individual lifestyle choices. We assess the share of health inequality explained by these determinants in the SSA countries included in our study, using the standardized BMI as a health indicator (Fig. 1). These estimates are based on three inequality indices: the variance, the Gini coefficient and the MLD.

Consistent with prior research, the Gini index yields substantially higher estimates of health inequality [25,55]. This occurs because the Gini index is particularly sensitive to disparities near the center of the distribution, whereas variance captures differences more strongly among obese and extremely thin individuals, and the MLD primarily reflects inequalities at the lower end of the distribution. As explained in Section 2, our inequality estimates are based on differences in average BMI across groups of individuals. This approach results in a smoothed distribution that eliminates extreme values, which would otherwise have a greater impact on the MLD and variance.

The Gini index for illegitimate factors ranges from 0.1635 in Egypt (2014) to 0.5436 in Kenya (2022), while variance values range from 0.0365 to 0.2839 and MLD estimates from 0.0429 to 0.2732 in the same countries. Despite differing levels across indices, the country rankings remain relatively stable, with Kenya, Eswatini and Mauritania showing the largest values.

**Fig. 2:**
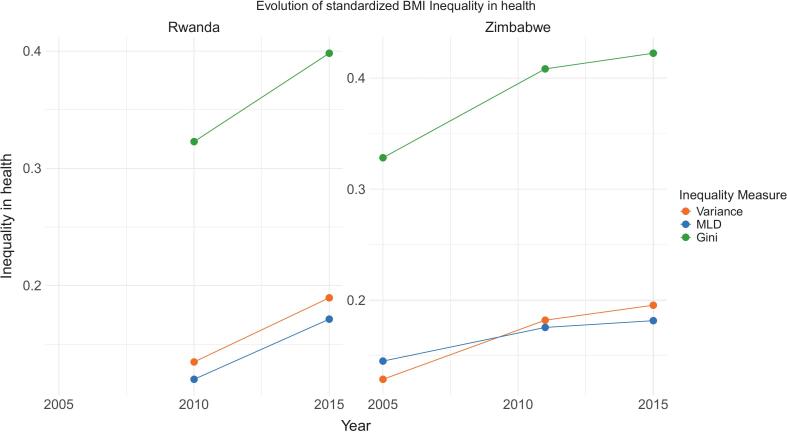
Evolution of health inequality in Rwanda and Zimbabwe. .

Source: Authors’ compilation. Note: Health inequality has been estimated using conditional inference-tuned trees, with the optimal parameters detailed in Table A4. Inequality in health is expressed as a proportion of the total variation in BMI (Body Mass Index)

For countries with multiple survey years, we tracked health inequality over time. Fig. 2 shows these trends for Rwanda (2010–2015) and Zimbabwe (2005–2015). Zimbabwe experienced significant increases in inequality: approximately 10 percent based on the Gini index and 7 and 5 percent based on variance and MLD respectively. Rwanda shows a similar pattern with a 7 percent increase by Gini measures and 5 percent by variance and MLD. These trends suggest that policies have failed to reduce structural health disparities, potentially worsening existing inequalities.

### 4.2 Determinants of health inequality

**Fig. 3:**
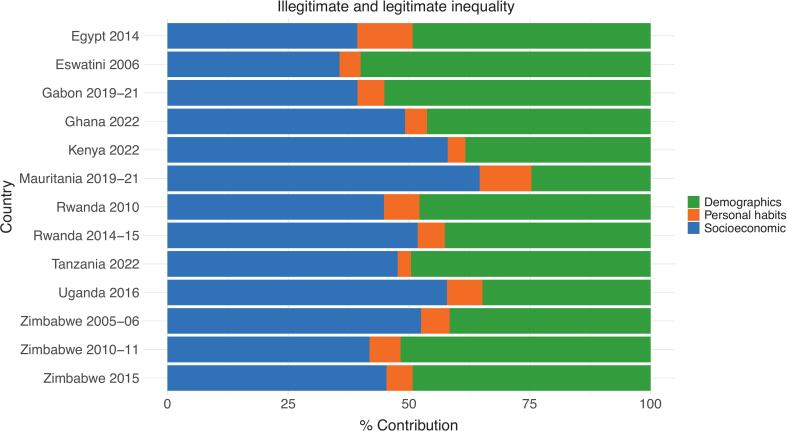
Contribution of legitimate and illegitimate factors to differences in BMI (Body Mass Index). Variance. . Note: The contribution of each set of variables has been estimated using the Shapley-Shorrocks value decomposition method described in Section 2.

Source: Authors’ compilation

Having established the overall patterns of health inequality across countries, we now examine the specific factors that drive these disparities. We apply Shapley decomposition to estimate the contribution of individual determinants to health disparities. Fig. 3 presents the contribution to health inequality of socioeconomic factors (asset ownership, wealth, and education), demographic aspects (age and gender), and legitimate determinants (tobacco consumption and healthy dietary habits).

Socioeconomic factors account for approximately 50 percent or more of health disparities in Ghana, Kenya, Mauritania, Rwanda (2014/15), Uganda, and Zimbabwe (2005/06). In contrast, demographic determinants explain 50 percent or more of the observed health inequality in Egypt, Eswatini, Gabon, Tanzania, and Zimbabwe (2010–2015). In Rwanda (2010), no single category dominates health inequality. Socioeconomic factors contribute approximately 45 percent, demographic variables account for about 47 percent, and lifestyle choices represent 7 percent of BMI differences.

Notably, the impact of legitimate lifestyle choices on overall inequality remains minimal across all cases, ranging from 3 percent in Tanzania to 11 percent in Egypt. These findings suggest that, although individual behaviors contribute to health disparities, their overall influence is marginal compared to structural determinants such as socioeconomic status and demographic factors. Consequently, policy efforts aimed at reducing health inequality should primarily focus on addressing systemic socioeconomic and demographic disparities rather than solely promoting behavioral changes.

We now examine the impact of each variable to BMI differences (Table 1). Among socioeconomic factors, wealth and education consistently emerge as most influential across all countries. Wealth accounts for the largest share of inequality, with contributions reaching 25.84 percent in Mauritania and 21.34 percent in Ghana. Education’s impact ranges from 13.11 percent in Mauritania to 20.71 percent in Egypt (2014). These findings highlight how financial stability and educational attainment link to better health outcomes through improved healthcare access, nutrition, and living conditions.

**Table 1:** Contribution of individual determinants to BMI (Body Mass Index) variation by country. Variance.

Country	Residence	Car	TV	Cellphone	Wealth	Education	Total socioeconomic	Sex	Age	Total demographic	Tobacco	Vegetables	Total legitimate
Egypt 2014	3.62	2.39	0.11	0.45	12.02	20.71	39.30	7.29	41.98	49.27	6.60	4.82	11.42
Eswatini 2006	1.93	2.34	2.36	3.73	9.48	15.79	35.63	16.52	43.49	60.01	1.10	3.27	4.37
Gabon 2019–21	2.85	1.40	1.58	0.51	13.63	19.36	39.33	16.72	38.41	55.13	0.85	4.71	5.56
Ghana 2022	5.56	2.63	4.51	0.12	21.34	15.02	49.18	15.20	31.08	46.28	0.66	3.88	4.54
Kenya 2022	4.72	1.75	11.79	1.13	21.18	17.41	57.98	11.26	27.08	38.34	0.91	2.77	3.68
Mauritania 2019–21	5.93	12.91	5.94	0.88	25.84	13.11	64.61	2.30	22.39	24.69	9.67	1.03	10.70
Rwanda 2010	3.84	0.06	4.27	3.99	13.87	18.78	44.81	16.06	31.77	47.83	3.13	4.24	7.37
Rwanda 2014–15	4.71	0.16	9.65	6.65	13.44	17.18	51.79	16.00	26.58	42.58	1.18	4.44	5.62
Tanzania 2022	5.81	0.63	5.20	1.00	19.62	15.39	47.65	9.89	39.72	49.61	0.49	2.25	2.74
Uganda 2016	4.31	1.95	6.31	6.08	20.29	18.94	57.88	6.63	28.16	34.79	0.57	6.76	7.33
Zimbabwe 2005–06	5.91	2.56	7.74	3.97	16.69	15.62	52.49	4.50	37.09	41.59	0.57	5.35	5.92
Zimbabwe 2010–11	4.84	2.22	3.76	4.54	11.58	14.88	41.82	15.91	35.82	51.73	3.19	3.25	6.44
Zimbabwe 2015	3.96	4.53	6.47	2.87	13.87	13.61	45.31	12.98	36.27	49.25	0.02	5.41	5.43

Source: Authors’ compilation.

Note: The contribution of each set of variables has been estimated using the Shapley-Shorrocks value decomposition method described in Section 2.

Ownership of durable goods also contributes to health inequality, though less significantly. In some countries, certain assets have disproportionate impact, such as TV ownership in Kenya (11.79 percent) or car ownership in Mauritania (12.91 percent). Geographic residence (urban vs. rural) is another key factor in countries like Mauritania (5.93 percent), Tanzania (5.81 percent), and Zimbabwe (5.91 percent), where rural populations face greater barriers to healthcare and nutritional diversity.

For demographic factors, since BMI has been standardized to eliminate physiological variations by age and sex, observed differences reflect non-biological influences like societal norms. Age has the strongest impact, contributing between 43.49 percent (Eswatini) and 22.39 percent (Mauritania). Gender’s role is comparatively smaller, accounting for approximately 16 percent of BMI variation in six countries, but as little as 2.3 percent in Mauritania, 6.63 percent in Uganda, and 4.5 percent in Zimbabwe (2005). These findings suggest that while age-related disparities significantly shape health inequality, gender-based differences vary considerably across countries, reflecting cultural and social influences.

Legitimate factors account for only a small share of health inequality, typically between 4 and 7 percent. Egypt and Mauritania show the largest impact of lifestyle choices (11.42 and 10.70 percent, respectively), with tobacco consumption being the main driver. In the other countries, vegetable consumption emerges as the predominant factor shaping legitimate differences in BMI.

## 5 Conclusions

This paper provides a comprehensive analysis of health inequalities in ten countries in the SSA region using the BMI as a health metric. We rely on nationally representative data and machine-learning methodologies to examine the structural factors driving health disparities. Our findings highlight the multifaceted nature of health inequality, with socioeconomic factors, predominantly wealth and education, and demographic characteristics emerging as the primary contributors. In contrast, lifestyle choices, such as smoking and dietary habits, appear to have a minimal impact on health disparities. These results underscore the significant role of structural determinants of health, reinforcing the argument that reducing inequality requires addressing its root causes, such as limited economic resources. Additionally, our estimates suggest that gender is also a relevant driver of health inequality, which reflects deep-rooted societal norms and systemic biases that disproportionately affect women.

The analysis of health inequality trends in Rwanda and Zimbabwe reveals alarming increases in both countries. Despite global efforts to reduce health disparities [56,57,58], structural barriers persist and, in some cases, have worsened. These findings suggest that current health and social policies have been insufficient in addressing systemic inequities, particularly among marginalized populations. This underscores the urgent need for more effective strategies to combat health inequality in these countries.

Our analysis has three key limitations. First, standardized BMI may not fully capture all health aspects as it does not account for variations in body composition. Second, our analysis omits important variables like healthcare access, which Bommier and Stecklov [59] identify as crucial for distinguishing between illegitimate disparities and those addressable through policy interventions. Unfortunately, DHS surveys lack healthcare access data. Third, we include only two legitimate determinants (vegetable consumption and smoking) while considering numerous illegitimate factors. This might potentially underrepresent the contribution of individual behaviors to health inequality.

Despite these limitations, our findings have clear policy implications. Given that socioeconomic factors account for 40–65 percent of health inequality, policies should prioritize structural economic interventions. These should include expanding social protection programs that provide support to low-income households, progressive taxation systems that can fund universal health coverage initiatives, and investing in quality primary education. The substantial contribution of wealth inequality suggests that economic development strategies, including job creation programs and rural development projects, may yield significant health benefits.

The role of geographic disparities (2–6 percent of inequality) requires financial incentives for healthcare workers to practice in rural locations, and investments in rural infrastructure. Gender’s significant contribution (up to 16 percent) requires interventions that focus on eliminating financial barriers that disproportionately affect women’s healthcare access, and implementing programs that address cultural and social barriers to women’s healthcare access. In addition, while lifestyle factors contribute modestly (3–11 percent), policies should address structural determinants of healthy choices rather than focusing solely on individuals’ behavioral change. However, these recommendations require adaptation to each country’s contexts.

## CRediT authorship contribution statement

**Mercedes Tejería-Martínez:** Writing – review & editing, Writing – original draft, Visualization, Validation, Supervision, Software, Resources, Project administration, Methodology, Investigation, Formal analysis, Data curation, Conceptualization. **Vanesa Jordá:** Writing – review & editing, Writing – original draft, Visualization, Validation, Supervision, Software, Resources, Project administration, Methodology, Investigation, Funding acquisition, Formal analysis, Data curation, Conceptualization. **José María Sarabia:** Writing – review & editing, Writing – original draft, Visualization, Validation, Supervision, Software, Resources, Project administration, Methodology, Investigation, Formal analysis, Data curation, Conceptualization.

## Funding

The authors acknowledge partial financial support from the I+D+i project ref. PID2024-156871NB-I00 finance by MICIU/AEI/10.1309/501100011033/FEDER,UE. Vanesa Jordá and Mercedes Tejería-Martínez also report partial support from Ramón Areces Foundation (Project CISP20A6658).

## Declaration of competing interest

The authors declare the following financial interests/personal relationships which may be considered as potential competing interests: Vanesa Jorda and Mercedes Tejeria-Martinez report financial support was provided by Ramon Areces Foundation. Vanesa Jorda and Mercedes Tejeria-Martinez report financial support was provided by Government of Spain, Ministry of Science, Innovation and Universities. Jose Maria Sarabia reports financial support was provided by Government of Spain, Ministry of Science, Innovation and Universities. If there are other authors, they declare that they have no known competing financial interests or personal relationships that could have appeared to influence the work reported in this paper.

## Footnotes

1 While we acknowledge the valuable body of existing research on health inequalities, including some contributions in low- and middle-income countries, our statement refers specifically to the limited availability of comprehensive, nationally representative data that allows the analysis of health inequality in sub-Saharan Africa. Most existing research focuses on single countries or single dimensions of inequality (typically income-related) (see e.g. [[60], [61], [62]], while comprehensive analyses incorporating multiple determinants affecting health disparities remain scarce for the SSA region.

2 While standardized BMI serves as a valuable proxy for nutritional status and metabolic risk, it does not distinguish between muscle mass and fat mass or capture other important health dimensions such as cardiovascular fitness or mental health. These limitations are discussed further in the conclusion.

3 The sample size of 1,003 corresponds to the smallest dataset in our study.
