# Supplementary material for: Structural drivers of health inequality in sub-Saharan Africa: Evidence and policy implications
Source: Health Policy Open. 2025 Nov 5;10:100151. doi: 10.1016/j.hpopen.2025.100151 (PMC12662074; doi:10.1016/j.hpopen.2025.100151)
Supplement: Supplementary Data 1 [file mmc1.docx]

**Supplementary material – Structural Drivers of Health Inequality: Evidence from 10 Sub-Saharan Countries**

**Table A1.** Proportion of Muslim population in Sub Saharan Africa countries.

| **Country** | **Muslim population** |
| --- | --- |
| Egypt | 90% |
| Eswatini | 2% |
| Gabon | 10.8% |
| Ghana | 19.9% |
| Kenya | 10.9% |
| Mauritania | 100% |
| Rwanda | 2.1% |
| Tanzania | 34.1% |
| Uganda | 13.7% |
| Zimbabwe | 1.5% |

Source: Central Intelligence Agency, 2024.

**Table A2.** Summary statistics of standardized BMI.

| **Country** | **Sample size** | **Mean** | **Std. Dev.** | **Var.** | **Gini** | **MLD** |
| --- | --- | --- | --- | --- | --- | --- |
| Egypt 2014 | 14426 | 28.51 | 4.8219 | 23.2504 | 0.0931 | 0.0137 |
| Eswatini 2006 | 1003 | 25.70 | 4.3996 | 19.3567 | 0.0955 | 0.0140 |
| Gabon 2019-21 | 1027 | 24.88 | 4.3151 | 18.6202 | 0.0968 | 0.0144 |
| Ghana 2022 | 1476 | 23.67 | 3.6920 | 13.6309 | 0.0875 | 0.0118 |
| Kenya 2022 | 4151 | 22.77 | 4.2436 | 18.0084 | 0.1039 | 0.0167 |
| Mauritania 2019-21 | 1446 | 28.11 | 6.0872 | 37.054 | 0.1215 | 0.0228 |
| Rwanda 2010 | 1282 | 22.14 | 2.5080 | 6.2902 | 0.0625 | 0.0062 |
| Rwanda 2014-15 | 1450 | 22.68 | 2.7860 | 7.7615 | 0.0672 | 0.0072 |
| Tanzania 2022 | 1704 | 22.69 | 2.8661 | 8.2144 | 0.0722 | 0.0080 |
| Uganda 2016 | 1436 | 22.25 | 3.0362 | 9.2182 | 0.0750 | 0.0089 |
| Zimbabwe 2005-06 | 1816 | 23.00 | 3.4112 | 11.6363 | 0.0813 | 0.0104 |
| Zimbabwe 2010-11 | 1611 | 22.81 | 3.2126 | 10.3208 | 0.0797 | 0.0097 |
| Zimbabwe 2015 | 1670 | 23.57 | 3.5687 | 12.7356 | 0.0843 | 0.0110 |

Source: Authors’ compilation.

**Table A3.** Summary statistics of socioeconomic and demographic variables.

| Country |  | Egypt 2014 | Eswatini 2006 | Gabon  2019-21 | Ghana 2022 | Kenya 2022 | Mauritania 2019-21 | Rwanda 2010 | Rwanda 2014-15 | Tanzania 2022 | Uganda 2016 | Zimbabwe 2005-06 | Zimbabwe 2010-11 | Zimbabwe 2015 |
| --- | --- | --- | --- | --- | --- | --- | --- | --- | --- | --- | --- | --- | --- | --- |
| **Sex** | Female | 7254 | 737 | 719 | 977 | 2689 | 967 | 752 | 879 | 1095 | 914 | 1283 | 1093 | 1178 |
|  | Male | 7172 | 266 | 308 | 499 | 1462 | 479 | 530 | 571 | 609 | 522 | 533 | 518 | 492 |
| **Age** | Mean age (in years) | 31.29 | 30.22 | 31.84 | 31.85 | 30.57 | 33.02 | 30.64 | 30.86 | 30.93 | 30.11 | 29.04 | 29.59 | 30.15 |
| **Car** | No | 13029 | 809 | 919 | 1369 | 3893 | 1219 | 1265 | 1421 | 1656 | 1369 | 1724 | 1498 | 1451 |
|  | Yes | 1397 | 194 | 108 | 107 | 258 | 227 | 17 | 29 | 48 | 67 | 92 | 113 | 219 |
| **TV** | No | 263 | 644 | 189 | 557 | 2416 | 837 | 1158 | 1206 | 1656 | 1183 | 1286 | 985 | 894 |
|  | Yes | 14163 | 359 | 838 | 919 | 1735 | 609 | 124 | 244 | 515 | 253 | 530 | 626 | 776 |
| **Cellphone** | No | 657 | 378 | 47 | 83 | 279 | 157 | 683 | 497 | 209 | 329 | 16574 | 566 | 180 |
|  | Yes | 13769 | 625 | 980 | 1393 | 3872 | 1289 | 599 | 953 | 1495 | 1107 | 242 | 1045 | 1490 |
| **Wealth Index** | Poorest | 2319 | 184 | 405 | 382 | 1312 | 331 | 246 | 321 | 334 | 354 | 383 | 348 | 333 |
|  | Poorer | 2668 | 216 | 202 | 346 | 705 | 231 | 287 | 257 | 266 | 284 | 361 | 300 | 252 |
|  | Middle | 3128 | 191 | 180 | 274 | 680 | 351 | 221 | 239 | 328 | 249 | 304 | 278 | 226 |
|  | Richer | 3121 | 197 | 117 | 260 | 823 | 287 | 210 | 243 | 406 | 270 | 450 | 367 | 485 |
|  | Richest | 3190 | 215 | 123 | 214 | 63º | 246 | 318 | 390 | 370 | 279 | 318 | 318 | 374 |
| **Type of residence** | Urban | 5865 | 281 | 780 | 681 | 1464 | 688 | 256 | 415 | 550 | 304 | 631 | 593 | 706 |
|  | Rural | 8561 | 722 | 247 | 795 | 2687 | 758 | 1026 | 1359 | 1154 | 1132 | 1185 | 1018 | 964 |
| **Education** | Mean maximum education years achieved | 9.291 | 8.016 | 8.5220 | 7.392 | 7.9740 | 3.73 | 4.354 | 5.121 | 7.199 | 6.771 | 8.128 | 9.366 | 9.717 |

Source: Authors’ compilation.

**Table A4.** Summary statistics of legitimate inequality variables.

| Country |  | Egypt 2014 | Eswatini 2006 | Gabon  2019-21 | Ghana 2022 | Kenya 2022 | Mauritania 2019-21 | Rwanda 2010 | Rwanda 2014-15 | Tanzania 2022 | Uganda 2016 | Zimbabwe 2005-06 | Zimbabwe 2010-11 | Zimbabwe 2015 |
| --- | --- | --- | --- | --- | --- | --- | --- | --- | --- | --- | --- | --- | --- | --- |
| **Tobacco** | Number of cigarettes smoked | 0.5297 | 0.1426 | 0.2746 | 0.04946 | 0.1841 | 1.013 | 0.3112 | 0.211 | 0.1326 | 0.2911 | 0.3888 | 0.6089 | 0.3503 |
| **Childveg** | No | 11191 | 554 | 745 | 1121 | 3120 | 1330 | 717 | 773 | 1477 | 1047 | 1387 | 1084 | 1021 |
|  | Yes | 3235 | 449 | 282 | 355 | 1031 | 116 | 565 | 677 | 227 | 389 | 449 | 527 | 649 |

Source: Authors’ compilation.

**Table A5.** Mean number of groups in each country.

| **Country** | **Groups** |
| --- | --- |
| Egypt 2014 | 2 |
| Eswatini 2006 | 10 |
| Gabon 2019-21 | 10 |
| Ghana 2022 | 9 |
| Kenya 2022 | 10 |
| Mauritania 2019-21 | 10 |
| Rwanda 2010 | 7 |
| Rwanda 2014-15 | 5 |
| Tanzania 2022 | 11 |
| Uganda 2016 | 9 |
| Zimbabwe 2005-06 | 4 |
| Zimbabwe 2010-11 | 9 |
| Zimbabwe 2015 | 9 |

Source: Authors’ compilation.

**Table A6.** Health inequality estimates.

| **Country** | **Variance** | **MLD** | **Gini** |
| --- | --- | --- | --- |
| Egypt 2014 | 0.0365 | 0.0429 | 0.1635 |
|  | [0.0179-0.0696] | [0.0160-0.0789] | [0.0955-0.2537] |
| Eswatini 2006 | 0.2779 | 0.2847 | 0.5149 |
|  | [0.2174-0.3436] | [0.2275-0.3471] | [0.4557-0.5675] |
| Gabon 2019-21 | 0.1497 | 0.196 | 0.3966 |
|  | [0.0956-0.2113] | [0.1175-0.2757] | [0.3018-0.4757] |
| Ghana 2022 | 0.2314 | 0.2733 | 0.4962 |
|  | [0.1817-0.2962] | [0.2000-0.3387] | [0.4278-0.5679] |
| Kenya 2022 | 0.2892 | 0.2929 | 0.5436 |
|  | [0.2411-0.3528] | [0.2131-0.3685] | [0.4752-0.6142] |
| Mauritania 2019-21 | 0.2839 | 0.2732 | 0.5111 |
|  | [0.2313-0.3405] | [0.2157-0.3372] | [0.4589-0.5832] |
| Rwanda 2010 | 0.1349 | 0.1201 | 0.3228 |
|  | [0.0791-0.1991] | [0.0614-0.1782] | [0.2476-0.3905] |
| Rwanda 2014-15 | 0.1896 | 0.1714 | 0.3982 |
|  | [0.1318-0.2566] | [0.1143-0.2286] | [0.3408-0.4597] |
| Tanzania 2022 | 0.1762 | 0.1642 | 0.3905 |
|  | [0.1252-0.2312] | [0.1115-0.2241] | [0.3292-0.4616] |
| Uganda 2016 | 0.1923 | 0.2118 | 0.4386 |
|  | [0.1315-0.2532] | [0.1467-0.2844] | [0.3644-0.5148] |
| Zimbabwe 2005-06 | 0.1288 | 0.1452 | 0.3283 |
|  | [0.0758-0.1852] | [0.0990-0.1940] | [0.2578-0.4000] |
| Zimbabwe 2010-11 | 0.182 | 0.1755 | 0.4083 |
|  | [0.1259-0.2419] | [0.1165-0.2336] | [0.3325-0.4729] |
| Zimbabwe 2015 | 0.1955 | 0.1816 | 0.4224 |
|  | [0.1463-0.2611] | [0.1252-0.2478] | [0.3638-0.4883] |

Source: Authors’ compilation.

**Table A7.** Optimal parameter combinations of hyperparameters for conditional inference trees. Variance.

| **Country** | **RMSE** | $\text{α}$ |
| --- | --- | --- |
| Egypt 2014 | 4.5643 | 0.01 |
| Eswatini 2006 | 3.9538 | 0.05 |
| Gabon 2019-21 | 3.9852 | 0.10 |
| Ghana 2022 | 3.9827 | 0.10 |
| Kenya 2022 | 3.9780 | 0.05 |
| Mauritania 2019-21 | 5.0694 | 0.10 |
| Rwanda 2010 | 3.9592 | 0.10 |
| Rwanda 2014-15 | 3.9875 | 0.10 |
| Tanzania 2022 | 4.0031 | 0.10 |
| Uganda 2016 | 3.9918 | 0.01 |
| Zimbabwe 2005-06 | 4.0139 | 0.01 |
| Zimbabwe 2010-11 | 4.0017 | 0.10 |
| Zimbabwe 2015 | 3.9455 | 0.05 |

Source: Authors’ compilation.

**References**

Central Intelligence Agency (2024). *The World Factbook*.  Available at https://www.cia.gov/the-world-factbook/
